# Germline single nucleotide polymorphisms in *ERBB3* and *BARD1* genes result in a worse relapse free survival response for HER2-positive breast cancer patients treated with adjuvant based docetaxel, carboplatin and trastuzumab (TCH)

**DOI:** 10.1371/journal.pone.0200996

**Published:** 2018-08-02

**Authors:** Damien Coté, Alex Eustace, Sinead Toomey, Mattia Cremona, Malgorzata Milewska, Simon Furney, Aoife Carr, Joanna Fay, Elaine Kay, Susan Kennedy, John Crown, Bryan Hennessy, Stephen Madden

**Affiliations:** 1 Royal College of Surgeons in Ireland, Data Science Centre, Dublin, Ireland; 2 Dublin City University, National Institute for Cellular Biotechnology, Molecular Therapeutics for Cancer in Ireland, Dublin, Ireland; 3 Royal College of Surgeons in Ireland, Department of Molecular Medicine, Medical Oncology Group, Dublin, Ireland; 4 Royal College of Surgeons in Ireland, Department of Physiology and Medical Physics, Dublin, Ireland; 5 Royal College of Surgeons in Ireland, Department of Pathology, Dublin, Ireland; 6 St Vincent’s University Hospital, Medical Oncology, Dublin, Ireland; 7 Beaumont Hospital, Department of Medical Oncology, Dublin, Ireland; Fondazione IRCCS Istituto Nazionale dei Tumori, ITALY

## Abstract

Breast cancer is the leading cause of cancer related deaths in women worldwide and is classified into subtypes based on the cancer’s receptor status. Of these subtypes, those expressing the human epidermal growth factor receptor 2 (HER2) receptor were traditionally associated with poor prognosis. Several advances have been made in the treatment of HER2-positive breast cancer, yet issues of resistance and poor response to therapy remains prevalent. In this study we explored the impact of HER-family and homologous recombination deficiency SNPs on response to patients who received TCH-based (docetaxel (T), carboplatin (C), and trastuzumab (H)) treatment versus those who received other treatment regimens. Using Cox regression analysis, we identified 6 SNPs that correlate with recurrence free survival in our patients and supported our findings using support vector machines. We also used reverse phase protein array analysis to examine the impact ERBB3 SNPs may have on both the PI3K/AKT and MAPK/ERK signaling pathways. Finally, using cell line models, we correlated SNP status with sensitivity to platinum based drugs and docetaxel. We found that patients on a TCH based regimen with the minor allele of the ERBB3 (rs2229046 and rs773123) and BARD1 (rs2070096) SNPs, were significantly more likely to relapse than those women who were not. Additionally, we observed that patients with these ERBB3 SNPs had shown elevated protein expression/phosphorylation of Src kinase, c-MET (Y1234/1235), GSK-3β (S9) and p27, indicating that these SNPs are associated with non-PI3K/AKT signaling. Finally, using cell line models, we demonstrate that the BARD1 SNP (rs2229571) is associated with greater sensitivity to both carboplatin and cisplatin. The BARD1 and ERBB3 SNPs can potentially be used to determine those patients that will have a worse response to TCH based treatment, an effect that may arise from the SNPs impact on altered cellular signaling.

## Introduction

HER-2 positive breast cancer comprises cancers which exhibit the overexpression or amplification of the Erb-B2 Receptor Tyrosine Kinase 2 (*ERBB2*) gene (also known as the HER2 protein) and accounts for approximately 20% of all breast cancers. It is associated with a significantly worse survival [1,2]. Current adjuvant treatment strategies for women with HER2-positive breast cancer after their surgery include the use of trastuzumab (H), a humanized monoclonal antibody in combination with anthracyclines (A), taxanes (T) and platinum salts (C). Recent clinical studies have shown that combinations of ACT or ACTH or TCH have shown improved benefit in the adjuvant treatment of women with HER2-positive breast cancer, and are now the standard of care [3–6].

Recent data has suggested that germline single nucleotide polymorphisms (SNPs) play a role in both sensitivity and resistance to targeted therapies [7,8]. In fact, we and others have shown that SNPs which occur in the epidermal growth factor receptor (*EGFR*), *ERBB2*, *ERBB3* or *ERBB4* genes (HER-family genes) can be associated with either a worse relapse free survival (RFS) or worse overall survival (OS) rates in women with HER2-positive breast cancer who received adjuvant trastuzumab as part of their treatment regimen [9,10]. Mutations in HER-family genes have been shown to activate the PI3K/AKT signaling pathway and both germline SNPs and somatic mutations may act as biomarkers of sensitivity and resistance in both gastric and breast cancers [11].

Cancer cells with impairment in DNA repair mechanisms, such as homologous recombination deficiency (HRD), are sensitive to platinum based drugs, which directly damage a cell’s DNA. Mutations in the tumour suppressor genes *BRCA1/2* are the most common cause of HRD. However, any gene mutations which cause HRD can potentially result in a phenotype like that of *BRCA1/2* mutated cancer. This phenomenon is called ‘BRCAness’ and is also characterized by HRD [12].

Our current study aims to explore the effect on survival and functional impact of both HER-family and HRD related SNPs on response to TCH based treatment, versus patients who received a non-TCH based treatment.

## Materials and methods

### Patients

A total of 157 patients with operable primary BC were used in this study, which included 78 patient samples from the NCT01485926 phase II neo-adjuvant study of TCH/TCHL in women with early stage HER2-positive breast cancer. The remaining 79 patients came from the CTI-09-07 translational study, which enabled the collection of FFPE samples and the relevant clinicopathological data and treatment history. We selected an additional 32 samples from the CTI-09-07 translational study on which to perform high depth sequencing, as this discovery cohort had tumour blocks with sufficient tissue to perform NGS analysis. These studies were approved by the research ethics committees of all hospitals, were run by the All-Ireland Clinical Oncology Research Group (ICORG now Cancer Trials Ireland) and included women who are confirmed as clinically HER2-positive by a 3+ HER2 immunohistochemistry score and or/ a FISH ratio of >2. Detailed clinical information is available in Table 1. A Fisher exact test was used to compare the clinical parameters between TCH and non-TCH to show that there was no imbalance between the two cohorts. The p-values have been included in Table 1.

**Table 1:** Summary of patient characteristics (*n* = 157).

Feature	Sample Number	P-value
***TCH Samples (n = 102)***		
**Mean Age ± SD (years)**	51 ± 11	
**Grade**		
**I**	0	
**II**	10	
**III**	15	
**Unknown**	77	0.43
**LN Status**		
**Positive**	15	
**Negative**	14	
**Unknown**	73	0.86
**ER Status**		
**Positive**	62	
**Negative**	39	
**Unknown**	1	0.73
**PR Status**		
**Positive**	41	
**Negative**	54	
**Unknown**	7	0.07
**Mean OS ± SD (months)**	66 ± 59	
**Mean PFS ± SD (months)**	52 ± 33	
***Non TCH Samples (n = 55)***		
**Mean Age ± SD (years)**	53 ± 12	
**Grade**		
**I**	2	
**II**	15	
**III**	37	
**Unknown**	1	0.43
**LN Status**		
**Positive**	30	
**Negative**	24	
**Unknown**	1	0.86
**ER Status**		
**Positive**	31	
**Negative**	23	
**Unknown**	1	0.73
**PR Status**		
**Positive**	9	
**Negative**	27	
**Unknown**	19	0.07
**Mean OS ± SD (months)**	82 ± 35	
**Mean PFS ± SD (months)**	78 ± 37	

OS = Overall survival; PFS = Progression Free Survival; SD = Standard Deviation; LN = Lymph Node; ER = Estrogen Receptor; PR = Progesterone Receptor.

The patient data was analyzed anonymously, the study was approved by the ethics committee of all the individual hospitals in which the patients were treated, and approved the respective studies in accordance with the Declaration of Helsinki. CTI 09–07 is a translational study and enrolment on TCHL and CTI-0907 was possible. Central REC review for TCHL: University College Cork Clinical Research Ethics Committee, and Local REC reviews for 09–07: University College Cork Clinical Research Ethics Committee, Beaumont Hospital Ethics Committee, University Hospital Waterford Research Ethics Committee.

### High depth sequencing

In our initial SNP screen, we performed high depth sequencing on our discovery cohort, which contained 32 tumour formalin-fixed, paraffin-embedded (FFPE) samples, from patients with HER2-positive BC. Haematoxylin and eosin sections cut from the patient’s FFPE surgical blocks were analysed by a pathologist for tumour content and those with >50% tumour cellularity had a further 7*10μM sections cut, from which DNA was extracted using the Qiagen DDNA FFPE kit as outlined in a previous study [10].

### Sequencing analysis

The reads from our discovery cohort were trimmed using Trimmomatic [13] and aligned with BWA mem (version 0.7.5a-r405: http://bio-bwa.sourceforge.net/) under default parameters. Duplicate reads were marked by Picard tools (http://broadinstitute.github.io/picard/) and local realignment and base recalibration were conducted with GATK [14] (version v3.2-2-gec30cee, human genome version 19). Pileup files were generated using Samtools [15] (version 0.1.19-44428cd), excluding reads with mapping quality <20, and variants were called with Varscan [16] (version v2.3.7) at positions with coverage ≥20. Variants were annotated by Variant Effect Predictor [17].

### Protein extraction and reverse phase protein array analysis

We extracted protein from 60 women’s FFPE surgical samples and performed RPPA analysis to identify the impact of HER-family and HRD related SNPs on PI3K/AKT and MAPK/ERK signaling. We only selected FFPE samples from surgical tumour resections which had greater than 50% tumour or were enriched by macro dissection to ensure >50% tumour, and samples which had low protein yield were excluded from our analysis. RPPA on the 60 clinical samples was performed as previously described [18]. The data was normalized by protein loading using the entire antibody panel. These 60 samples are a subset of the original patient cohort. A breakdown of the clinical parameters of these samples is shown in Table 2. A Fisher exact test was used to compare the clinical parameters between TCH and non-TCH to show that there was no imbalance between the two cohorts and the p-values are shown in Table 2. The protein levels between TCH receiving patients and patients receiving another regiment were then compared using Tukey Honest Significant Difference and an ANOVA model. The resultant p-values are adjusted for multiple testing using the Benjamini-Hochberg method [19].

**Table 2:** Summary of patient characteristics used in the RPPA analysis (*n* = 60).

Feature	Sample Number	P-value
***TCH Samples (n = 27)***		
**Mean Age ± SD (years)**	50 ± 12	
**Grade**		
**I**	0	
**II**	10	
**III**	14	
**Unknown**	3	0.38
**LN Status**		
**Positive**	14	
**Negative**	13	
**Unknown**	0	0.80
**ER Status**		
**Positive**	18	
**Negative**	9	
**Unknown**	0	1.0
**PR Status**		
**Positive**	11	
**Negative**	11	
**Unknown**	5	
**Mean OS ± SD (months)**	65 ± 59	
**Mean PFS ± SD (months)**	59 ± 57	0.76
***Non TCH Samples (n = 33)***		
**Mean Age ± SD (years)**	53 ± 13	
**Grade**		
**I**	2	
**II**	9	
**III**	22	
**Unknown**	0	0.38
**LN Status**		
**Positive**	19	
**Negative**	14	
**Unknown**	0	0.80
**ER Status**		
**Positive**	21	
**Negative**	12	
**Unknown**	0	1.0
**PR Status**		
**Positive**	9	
**Negative**	13	
**Unknown**	11	0.76
**Mean OS ± SD (months)**	72 ± 34	
**Mean PFS ± SD (months)**	70 ± 36	

### Agena MassArray

Mass spectrometry-based genotyping (Agena MassARRAY, Sequenom, San Diego, CA) was applied to confirm the allele calls of the 6 SNPs which are listed in Table 3 from the NGS screen of HER-family and HRD related genes. We also tested the 157 HER2-positive BC patient cohort focusing only on the 6 most significant SNPs from the initial 32 sample screen. Reactions where >15% of the resultant mass ran in the mutant site were scored as positive.

**Table 3:** Impact of HER-family and HRD SNPs on relapse free and overall survival of HER2-positive BC patients who have received either TCH based regimen or a non-TCH based regimen as part of their therapeutic regimen (n = 157).

Gene Information				TCH Treated Patients		Patients Not Treated With TCH	
Gene	Accession Number	SNP	R/MA Sub	RFS HR (95% CI)	Adjusted p-value	RFS HR (95% CI)	Adjusted p-value
**ErbB2**	rs1136201	I655V	A/G	2.67 (1.05–6.78)	**0.05**	2.75 (0.80–9.52)	0.29
**ErbB3**	rs2229046	I449I	T/C	4.95 (1.91–2.79)	**1.51x10**^**-3**^	1.22 (0.26–5.77)	0.89
	rs773123	S1119C	T/A	2.67 (1.02–7.03)	**0.05**	0.65 (0.14–3.07)	0.89
**RNF8**	rs2284922	T448T	C/T	1.11 (0.24–5.06)	0.90	12.42 (2.00–77.19)	**0.01**
**BARD1**	rs2070096	T351T	G/T	3.27 (1.16–9.17)	**0.05**	0.89 (0.25–3.17)	0.89
	rs2229571	R378S	G/C	2.02 (0.66–6.13)	0.25	1.1 (0.28–4.26)	0.89

TCH = docetaxel, carboplatin, trastuzumab; R/MA = Reference/Minor Allele; RFS = Recurrence Free Survival; HR = Hazard Ratio; CI = Confidence Interval.

### Survival analysis

Survival analysis was only performed using data from trastuzumab treated samples, with RFS as the survival endpoint. Survival curves are based on Kaplan-Meier estimates and the log-rank p-value is shown for difference in survival. The resultant p-values are adjusted for multiple testing using the Benjamini-Hochberg method [19]. Cox regression analysis was used to calculate hazard ratios and perform multivariate analysis. The R package ‘survival’ is used to calculate and plot the Kaplan-Meier survival curves [20]. All calculations are carried out in the R statistical environment (http://cran.r-project.org/).

### Cell line analysis

Exome sequencing data for 70 breast cancer cell lines treated with 90 therapeutic agents [21] were downloaded from the gene expression omnibus (https://www.ncbi.nlm.nih.gov/geo/) in SRA format (accession number GSE48216). Samples were processed as per GATK [22] best practices (https://software.broadinstitute.org/gatk/best-practices/). Briefly, the SRA files were converted to FASTQ files using the SRA Toolkit (https://www.ncbi.nlm.nih.gov/sra/docs/toolkitsoft/), quality control was conducted using FastQC (http://www.bioinformatics.babraham.ac.uk/projects/fastqc/), the adapter sequences were trimmed using BBmap (http://jgi.doe.gov/data-and-tools/bbtools/bb-tools-user-guide/bbmap-guide/) and the FASTQ files were converted to SAM format using BWA [23]. Next, the duplicates were marked using Picard Tools (http://broadinstitute.github.io/picard/) and reordered before being passed on to GATK for base recalibration, using known sites as provided by the Broad Institute best practices guide. The files were then run through GATK Haplotype caller using discovery mode and recommended settings and the files were run through the GATK Variant Recalibrator, using the recommended settings for SNP discovery and known sites as provided by the Broad Institute best practices guide. Finally, the files were annotated using Oncotator [24].

For statistical analysis, the locus status of the mutations in each HER2-positive cell line was used to divide the cell lines into three groups (homogenous reference allele, heterogeneous, and homogenous minor allele). The GD_50_ values of the three groups were then compared using Tukey Honest Significant Difference and an ANOVA model. The GD_50_ values and the HER2 status of the cell line were as per the original authors [21]. This test was done for each SNP and each treatment of interest (carboplatin and cisplatin). A p-value of < 0.05 was considered significant. All p-values were adjusted using the Benjamini-Hochberg method [19].

### Machine learning methods

To perform pattern recognition analysis, a predictive model was built and tested using a Support Vector Machine (SVM). The features used were our 6 SNPs and the time to last follow-up (in months) to predict RFS with (1) or without (0) event and a radial-kernel SVM was built using the e1071 package in R [25]. 5-fold cross validation was used to combat the issues that are associated with training a SVM on a dataset of this size when building on the development set, and leave one out cross validation (LOOCV) was used on the test set. This provided a vector of predictions equal in length to the vector of true values for RFS, where the two were compared and the following metrics were reported; accuracy, AUC and Phi score.

The accuracy was chosen to represent the predictive power of the model, the Phi score was utilized to determine how strong of a relationship existed between features and the classes, and the AUC was chosen to assess how efficient the features are at separating the classes, where a value of 0.5 represents the machine just ‘guessing’ the classes. The Phi score is a metric with a value between -1 and 1, where -1 represents an inverse correlation between the features and the classes, 1 represents a strong positive correlation [26].

## Results

We focused on 6 SNPs identified in our high depth sequencing study of the exome of 28 genes (S1 Table) from DNA extracted from the FFPE tumour samples of 32 women with HER2-positive breast cancer due to their correlation with RFS (Table 4). Included in our analysis were 3 SNPs from *ERBB2* and *ERBB3* genes; the *ERBB2* rs1136201 SNP, the *ERBB3* rs2229046 and rs773123 SNPs, which were found in approximately 15%, less than 5%, and 6% of the 1000 genome UK population, respectively. Our analysis also included 3 SNPs related to HRD; *RNF8* rs2284922 SNP, found in approximately 44% of the 1000 genome UK population and *BARD1*’s rs2070096 and rs2229571 SNPs, which occur in approximately 19% and 45% of the 1000 genome population, respectively. All 6 SNPs are exonic and occur in protein coding domains, where 3 (rs2229046, rs2284922, and rs2070096) are nonsynonymous variations, with rs2229046 being implicated in alternative splicing for *HER3* [27]. The frequency of each genotype within our study population can be found in S2 Table.

**Table 4:** List of SNPs including Gene ID, accession number, mutant allele frequency and haplotype details which were detected by high depth next generation sequencing and selected for further analysis.

Gene	Accession Number	GMAF	R/MA Sub	Haplotype	AA Change	Protein Domain
HER2	rs1136201	0.1556	A/G	37879588 A>G	I655V	Transmembrane
HER3	rs2229046	0.0495	T/C	56487201 T>C	I449I	Receptor L domain
HER3	rs773123	0.0665	T/A	56494998 A>T	S1119C	No defined domain
RNF8	rs2284922	0.4397	C/T	37349033 G>A	T448T	No defined domain
BARD1	rs2070096	0.1903	G/T	215645545 C>A	T351T	No defined domain
BARD1	rs2229571	0.4593	G/C	215645464 C>G	R378S	No defined domain

GMAF = Global Minor Allele Frequency; R/MA = Reference/Minor Allele; AA = Amino Acid.

### Association between HER-family and HRD related polymorphisms with drug response in HER2-positive patients

In our study, we assessed the impact of HER-family and HRD SNPs on the response of patients who received TCH-based treatment versus those who received other standard treatment regimens which did not include TCH.

**Fig 1:**
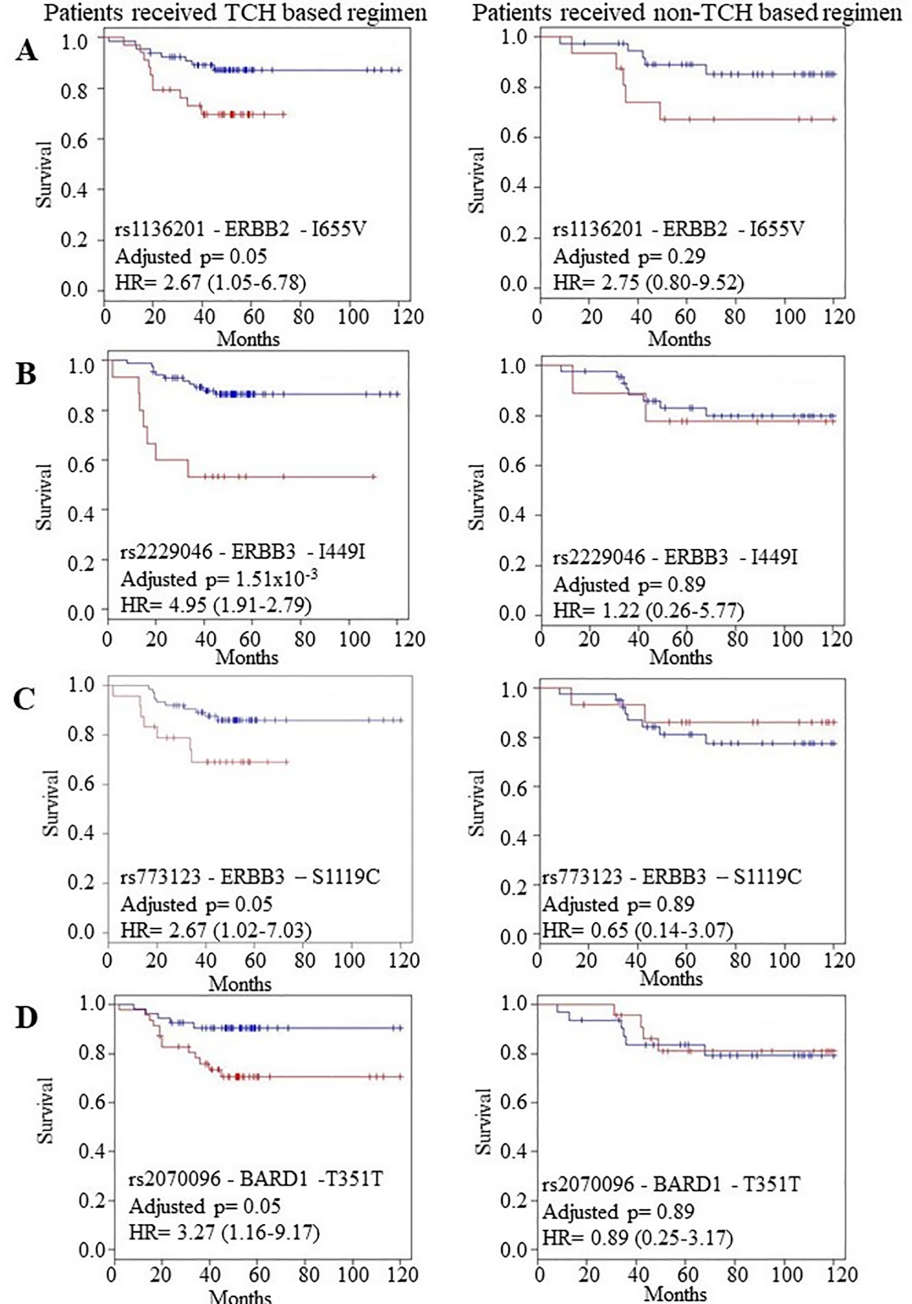
The prognostic role of ERBB2, ERBB3 and BARD1 SNPs in TCH versus non-TCH treated primary tumours (n = 157). In each plot, the blue and red lines represent the samples with the wild type (reference) and heterozygous alleles respectively. Kaplan Meier estimates of (A) the ERBB2-rs1136201 SNP where RFS is the survival endpoint (HR = 2.67 (1.05–6.78), p = 0.05) in TCH treated patients versus no-significant impact in non-TCH treated patients, (B) the ERBB3 rs2229046 SNP, where RFS is the survival endpoint (HR = 4.95 (1.91–2.79), p = 1.51x10-3) in TCH treated patients versus no significant impact on non-TCH treated patients, (C) the ERBB3 rs773123 SNP, where RFS is the survival endpoint (HR = 2.67 (1.02–7.03), p = 0.05) in TCH treated patients versus no significant impact on non-TCH treated patients, and (D) the BARD1 rs2070096 SNP, where RFS is the survival endpoint (HR = 3.27 (1.16–9.17), p = 0.05) in TCH treated patients versus no significant impact in non-TCH treated patients. p-values <0.05 indicates a significant p-value after multiple testing correction.

We found that women who were heterozygous for the *ERBB2* rs1136201 SNP and the *ERBB3* SNPs rs2229046 and rs773123, were significantly more likely to relapse on a TCH based regimen (rs1136201: p = 0.05, rs2229046: p = 1.51x10^-3^, rs773123: p = 0.05) than those women who received a non-TCH based treatment (Table 3, Fig 1A, 1B and 1C). After multivariate analysis, the difference in the rate of RFS for rs1136201 and rs2229046 remains significant when adjusted for age, estrogen receptor (ER) and progesterone receptor (PR) status (rs1136201: p = 0.01, rs2229046: p = 0.01), however, rs773123 did not remain significant when adjusted for the same parameters (p = 0.29).

**Fig 2:**
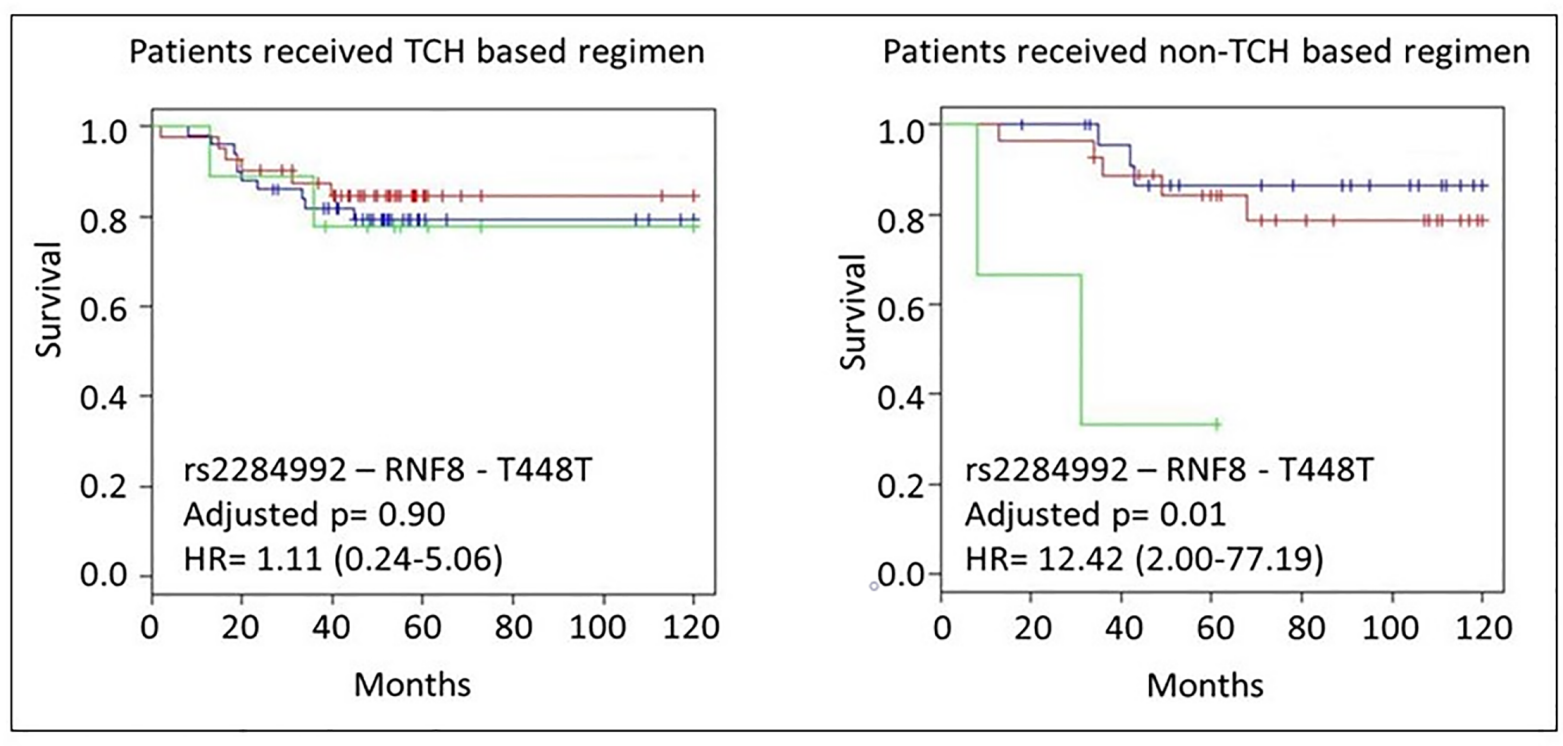
The prognostic role of RNF8-T448T SNP in TCH versus non-TCH treated primary tumours (n = 157). The blue, red and green lines represent the samples with the wild type (reference), heterozygous and minor alleles respectively. Kaplan Meier estimates where RFS is the survival endpoint (HR = 12.42 (2.00–77.19) p = 0.01) in non-TCH treated patients versus no-significant impact in TCH treated patients.

We also found that patients who were heterozygous for the *BARD1* rs2070096 SNP were significantly more likely to relapse on a TCH-based treatment than those patients (p = 0.05) who received a non-TCH based treatment (p = 0.89) (Fig 1D), which maintained its significance in RFS when adjusted for ER and PR status and age (p = 1.69x10^-4^). Conversely, we found that women who had the SNP for the *RNF8*, rs2284922, were significantly more likely to have a higher chance of relapse on non-TCH based treatment (p = 0.01), than those women who received TCH based treatment (p = 0.90) (Table 1, Fig 2). This maintains significance in RFS when adjusted for ER and PR status and age (p = 0.02).

### Cross-validation

Using machine learning, we demonstrate an increase in metric scores for support vector machine (SVM) model predicting RFS events in patients who underwent TCH based treatment versus those that received non-TCH based treatment (Table 5). A rise in these metrics reflects the increased ability of this model to predict RFS events in TCH vs non-TCH treated patients. Specifically, the Phi score shows a far stronger correlation between our SNPs and RFS in the TCH treated patients vs non-TCH treated patients.

**Table 5:** Metrics from the Support Vector Machine trained on the patient cohort using cross validation.

	Non-TCH	TCH
**Accuracy**	0.7962	0.8400
**Phi**	-0.0700	0.3600
**AUC**	0.4886	0.5789

TCH = docetaxel, carboplatin, trastuzumab; SVM = Support Vector Machine; AUC = Area Under the receiver-operator Curve

### *ERBB3* rs2229046 and rs773123 alter the signaling properties of human HER2-positive breast cancer cells

**Fig 3:**
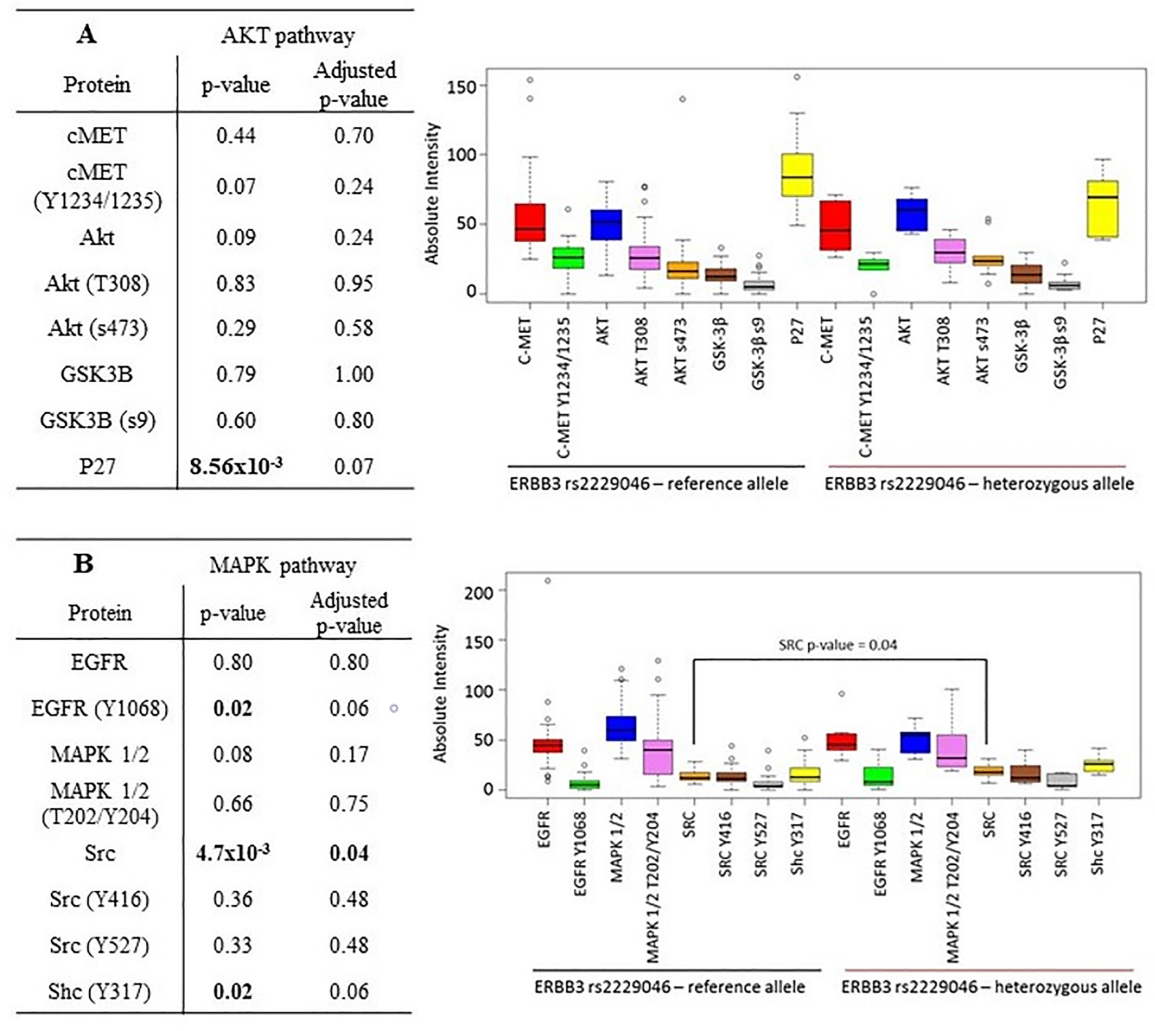
Reverse Phase Protein array analysis correlating differential expression and phosphorylation of proteins involved in either the A) AKT or B) MAPK pathway versus the presence or absence of the minor allele of the ERBB3 rs2229046 SNP. p-values <0.05 included on the graph demonstrate significantly differential protein expression between the presence of the reference allele or the presence of the minor allele and are corrected for multiple testing.

Our results indicated that patients who had the *ERBB3* rs2229046 SNP had a trend towards lower P27 expression (p-value = 0.07) but had significantly higher expression of SRC kinase (p-value = 0.04). These patients also had a trend towards higher phosphorylation of EGFR (Y1068) and Shc (Y317) (p-value = both 0.06). These results indicate that the *ERBB3* rs2229046 SNP may be associated with tumours which preferentially signal through the EGFR/MAPK/SRC kinase pathway (Fig 3).

**Fig 4:**
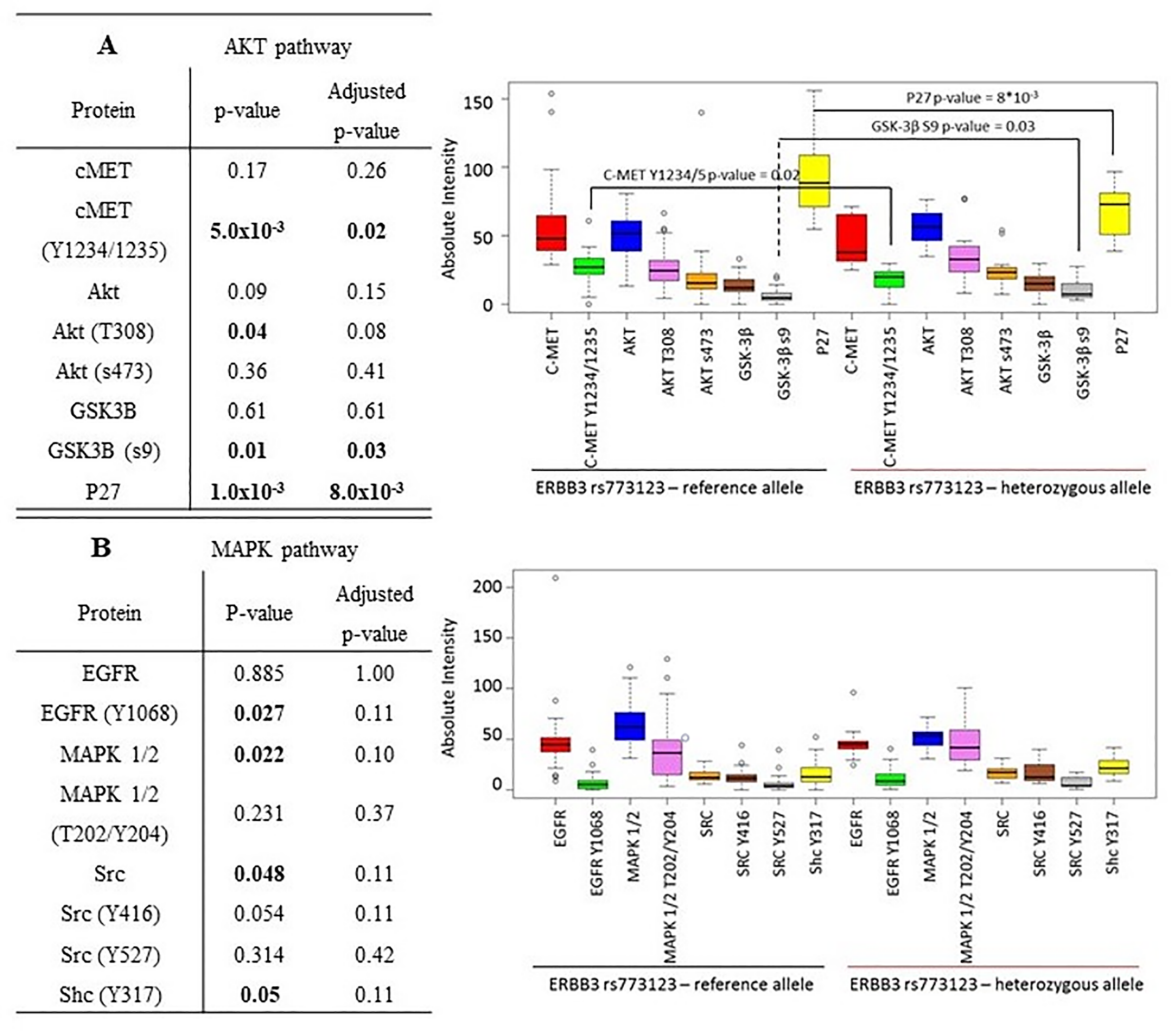
Reverse Phase Protein array analysis correlating differential expression and phosphorylation of proteins involved in either the A) AKT or B) MAPK pathway versus the presence or absence of the minor allele of the ERBB3 rs773123 SNP. p-values <0.05 included on the graph demonstrate significantly differential protein expression between the presence of the reference allele or the presence of the minor allele and are corrected for multiple testing.

We also found that patients who had the *ERBB3* rs773123 SNP were significantly more likely to have lower expression of P27 (Fig 4) (p-value 8.0x10^-3^), whilst also having significantly lower phosphorylation of c-MET (Y1234/1235) (p-value = 0.02) but significantly higher phosphorylation of GSK-3β (S9) (p-value = 0.03) (Fig 4). These results indicate that patients with the *ERBB3* rs773123 SNP may preferentially signal through the c-MET/AKT/GSK-3β pathway. However, reflecting the small size of this dataset (n = 60), it would be important to validate these findings in a larger dataset.

### The minor allele of *BARD1* rs2229571 is associated with higher sensitivity to platinum based drugs in in HER2-positive breast cancer cell lines

**Fig 5:**
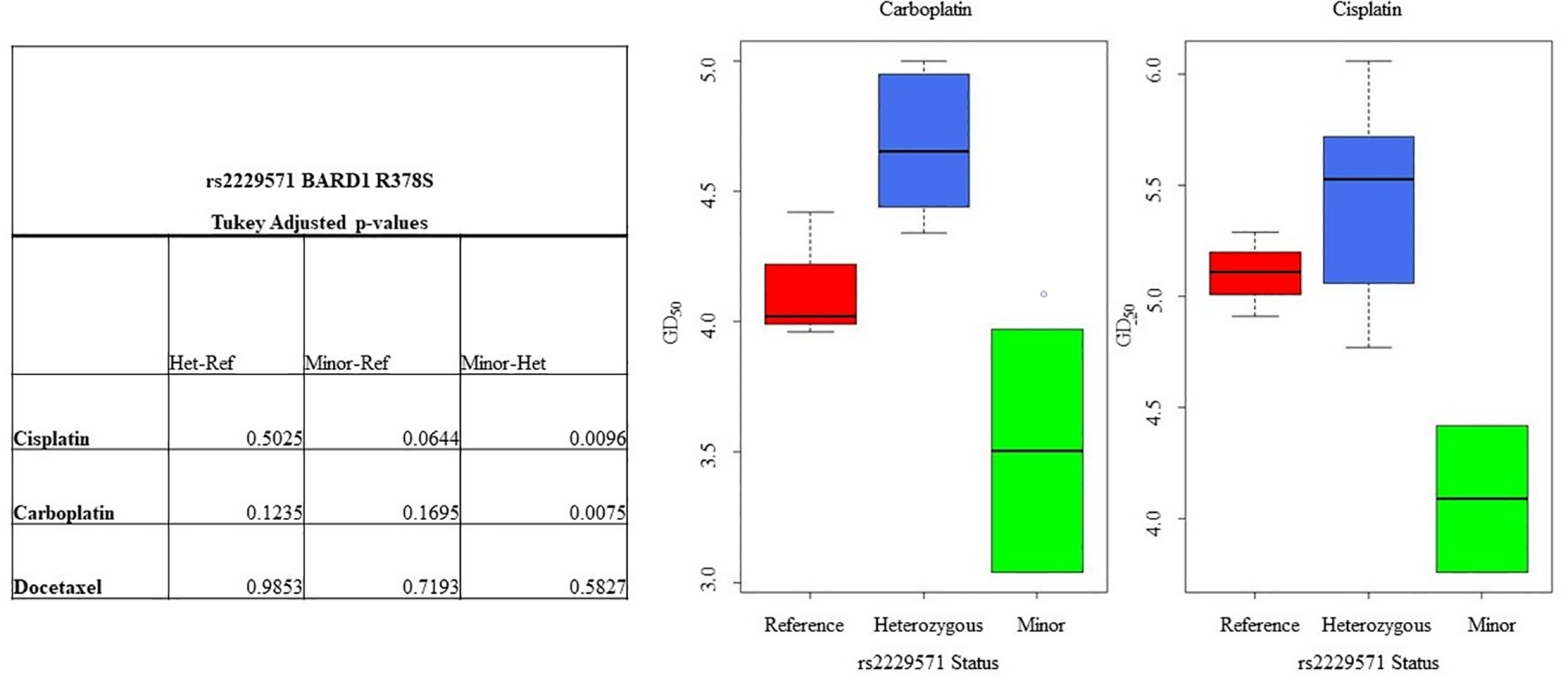
Analysis of the differential sensitivity of HER2-positive breast cancer cell lines to the platinum drugs cisplatin and carboplatin. We correlated the presence or absence of the minor allele of HER-family or BARD1 versus the GD_50_ to either drug. GD_50_ values were taken from the GDSC cell line analysis database [21]. p-value of <0.05 indicates a significant p-value after multiple testing correction.

Using available sequencing data from the Genomics of Drug Sensitivity in Cancer (GDSC) database [21] we determined the specific allele for each of the HER-family and HRD related SNPs in a panel of 11 HER2-positive cell lines (Table 6). We then correlated the sensitivity of the different cell lines based on the expression of the allele for each SNP against sensitivity to the platinum based drugs carboplatin and cisplatin, as well as docetaxel. Platinum and docetaxel based drugs form part of the TCH regimen used to treat women with HER2-positive breast cancer. Our results show that cell lines which had the minor allele of *BARD1* rs2229571 were significantly more likely to be sensitive to a platinum-based drug relative to those cell lines that had either the reference allele or were heterozygous for the SNP (carboplatin: p = 0.04, cisplatin: p = 0.02) (Fig 5). However, this result did not correlate with a difference in either RFS or OS in either TCH or non TCH treated patients. Furthermore, the same phenomena were not seen when we looked at breast cancer cell lines across multiple subtypes, demonstrating that this is unique to the HER2 enriched group (S1 File).

**Table 6:** The GD_50_ levels and SNP calls for the 11 HER2 Cell lines used as secondary validation in this study.

Cell Line	Carboplatin GD_50_	Cisplatin GD_50_	Docetaxel GD_50_	rs1136201 ERBB2 I655V	rs2229046 ERBB3 I449I	rs773123 ERBB3 S1119C	rs2284922 RNF8 T448T	rs2070096 BARD1 T351T	rs2229571 BARD1 R378S
AU565	4.95	5.72	8.30	Ref	Ref	Ref	Het	Ref	Het
BT474	3.97	4.42	8.20	Ref	Ref	Ref	Ref	Ref	Minor
HCC1419	4.02	4.91	7.80	Ref	Ref	Ref	Ref	Ref	Ref
HCC1569	3.96	5.11	8.12	Ref	Ref	Ref	Het	Ref	Ref
HCC1954	4.42	5.29	8.78	Ref	Ref	Ref	Minor	Ref	Ref
HCC202	4.44	6.06	8.63	Ref	Het	Het	Ref	Het	Het
MDAMB361	4.34	5.06	7.92	Ref	Ref	Ref	Ref	Ref	Het
SKBR3	5.00	4.77	8.00	Ref	Ref	Ref	Het	Ref	Het
UACC812	4.76	5.48	8.39	Ref	Ref	Ref	Het	Het	Het
UACC893	3.04	3.76	7.77	Ref	Het	Het	Ref	Ref	Minor
ZR7530	4.55	5.58	8.40	Ref	Ref	Ref	Het	Ref	Het

## Discussion

Despite the improvement in response rates of women with HER2-positive breast cancer to trastuzumab based treatments, a significant population of women have innate resistance to the trastuzumab based treatments such as the standard of care, TCH based therapy [2–5]. This innate resistance results in a clinically worse RFS for the patient. Currently there is no curative treatment for metastatic HER2-positive breast cancer; therefore, biomarkers which can identify at diagnosis patients unlikely to benefit from TCH based treatment are critical to allow clinicians to direct these patients to alternate therapeutic options.

Previously we and others demonstrated that the minor allele of the ERBB–I655V SNP was associated with a worse RFS in women with HER2-positive breast cancer [9,10]. A limitation of these studies was that a patient’s specific therapeutic regimens were not considered when the analysis was done. As trastuzumab is standardly prescribed in combination with either docetaxel or platinum based drugs (TCH) [3–6], we wanted to correlate SNPs as predictive markers of response to TCH based treatment versus other therapeutic regimens. In our study we not only looked at SNPs in the ERBB-family of genes but as the TCH regimen includes a platinum drug we also assessed the importance of SNPs in genes associated with HRD and correlated these differences with a patient’s response.

We have shown that patients who have the minor allele of the *ERBB3* SNPs, (rs2229046 and rs77123) and who received TCH based treatment are significantly more likely to have a worse RFS than patients who received alternate therapies. To support our hypothesis that this is a TCH dependent effect, we previously demonstrated that patients who had the minor allele of the *ERBB3* SNP rs2229046 and who received trastuzumab as part of their therapeutic regimen did not have a significantly worse RFS [10].

The *ERBB3* SNPs identified in our study represent both a synonymous (rs2229046) and non-synonymous (rs77123) SNP. SNPs which are synonymous variants were until recently believed to be silent, resulting in little to no impact on the ensuing protein. However, recent studies have shown that synonymous SNPs can play an important role in the functionality of the cancer cell and how a patient responds to targeted therapies [8,10,28,29].

Previous studies have shown that despite the *ERBB3* SNPs rs77123 and rs2229046 SNPs not having a described role in cancer susceptibility [30,31], both have been associated with alternative splicing [27]. To date though, no-one has analysed the impact of these SNPs on cellular signalling. Our results show that patients who have the minor allele of the *ERBB3* SNP rs2229046 have significantly elevated expression of Src kinase, and a nearly significant increase in EGFR Y1068 phosphorylation, whilst patients with the *ERBB3* SNP (rs77123) have a significantly higher level of GSK-3β (s9) phosphorylation relative to those with the reference allele. HER2-positive breast cancer is generally classified as being associated with activation of the PI3K/AKT signalling pathway [2,32,33]. Trastuzumab, the monoclonal antibody which targets HER2 is designed to block this PI3K/AKT activation by inhibiting downstream signalling of HER2 activation [2]. The elevated expression and phosphorylation of non-classically PI3K/AKT signalling pathways in cells that have minor allele of the *ERBB3* rs2229046 and rs77123 SNPs could potentially indicate why these patients do not respond as well to TCH based regimens relative to the patients who receive non-TCH based treatment.

Our analysis also identified that patients with the minor allele of the *BARD1* SNP rs2070096 were significantly more likely to have a worse RFS than those patients who received a non-TCH based treatment. Our hypothesis that impediment of the HRD pathway would result in altered sensitivity to platinum based drugs given as part of the TCH backbone is supported by our result that patients who were homozygous for the minor allele of *RNF8* rs2284992 had a worse RFS when they were given non-TCH based treatment, compared to those receiving TCH based treatment. The confounding effects of differing HRD related SNPs in relation to response to treatment would require further functional interrogation to elucidate why this effect occurs.

The role of RNF8 in DNA damage repair has been extensively studied, however there are no studies which associate *RNF8* rs2284922 as playing a role in cancer susceptibility or drug response [34]. However whilst both *BARD1* SNPs (rs2070096, rs2229571) in this study have been associated with nephroblastoma and neuroblastoma [35–38], no studies have associated them with response to platinum based drugs as part of a therapeutic regimen.

To further examine the impact of HRD related SNPs on platinum response we analysed the SNP status of a panel of 11 HER2-positive cell lines and correlated this against the cell line GD_50_ to platinum (cisplatin and carboplatin) and docetaxel based drugs. We demonstrated that those cell lines with the minor allele of the *BARD1* SNP rs2229571 had greater sensitivity to both carboplatin and cisplatin. Interestingly, the *BARD1* SNP rs2229571 had no significant effect on a patient’s response to TCH or non-TCH treatment. These results may indicate that whilst SNPs in HRD related genes do impact on a drug’s sensitivity in vitro, that their impact on a patient’s response clinically may be overcome using multiple therapies, which can have confounding effects on how a patient responds.

Our study does have limitations that must be taken into consideration, as these may lead to residual confounding biases. These issues include a small sample size and, in some cases, incomplete clinicopathological information. Future studies building from our work should test these variables to see if there are any residual effects on the results we have observed. One of the ways we addressed the small sample size in our study, was to perform rigorous statistical analysis to ensure that our results remain valid after correcting for multiple testing. We also used machine learning as another statistical tool to support that our patient response results are robust. Finally using the publicly available GDSC database we could determine the SNP status of cell line models which could be used to demonstrate the validity of our *BARD1* SNP results from patients in cell line models. We demonstrated that patients with the minor allele of the *ERBB3* and *BARD1* SNPs were more likely to have a worse RFS than those who received non-TCH based treatment. For the first time we also demonstrated using NGS analyses of cell line data that SNPs which occur in and *BARD1* correlate with carboplatin or cisplatin sensitivity in vitro. Finally, we have shown that patients with the minor allele of the *ERBB3* rs77123 and rs2229046 SNPs have elevated expression and phosphorylation of proteins in pathways not associated with PI3K/AKT signalling, likely indicating why these patients do worse to TCH based treatment.

This study has demonstrated the potential of SNPs, both synonymous and non-synonymous to be important markers in tumour response to therapy. These hereditary changes should be taken into account when clinicians decide on a suitable course of treatment for women who will receive adjuvant treatment of their HER2-positive breast cancer.

## Supporting information

S1 File Adjusted p-values for all the Tukey Honest Significant Difference tests and ANOVA models across the HER2 and the all the breast cancer cell lines. (CSV)

S1 Table The initial screen of 28 genes from DNA extracted from the FFPE tumour samples of 32 women with HER2-positive breast cancer. (CSV)

S2 Table The frequency of the 6 SNPs in the study population. (CSV)
